# Supplementary material for: Increase in concerns about climate change following climate strikes and civil disobedience in Germany
Source: Nat Commun. 2024 Apr 4;15:2916. doi: 10.1038/s41467-024-46477-4 (PMC10995135; doi:10.1038/s41467-024-46477-4)
Supplement: Supplementary file 1 — Supplementary Information [file 41467_2024_46477_MOESM1_ESM.pdf]

Supplementary Information for Increase in concerns about climate change following climate strikes and civil disobedience in Germany

Johannes Brehm[1,2] ✉ and Henri Gruhl[1,3] ✉

[1]RWI - Leibniz Institute for Economic Research, Berlin Office, Germany

[2]Hertie School, Berlin, Germany

[3]Vrije Universiteit Amsterdam, Amsterdam, Netherlands

✉ johannes.brehm@rwi-essen.de, henri.gruhl@rwi-essen.de

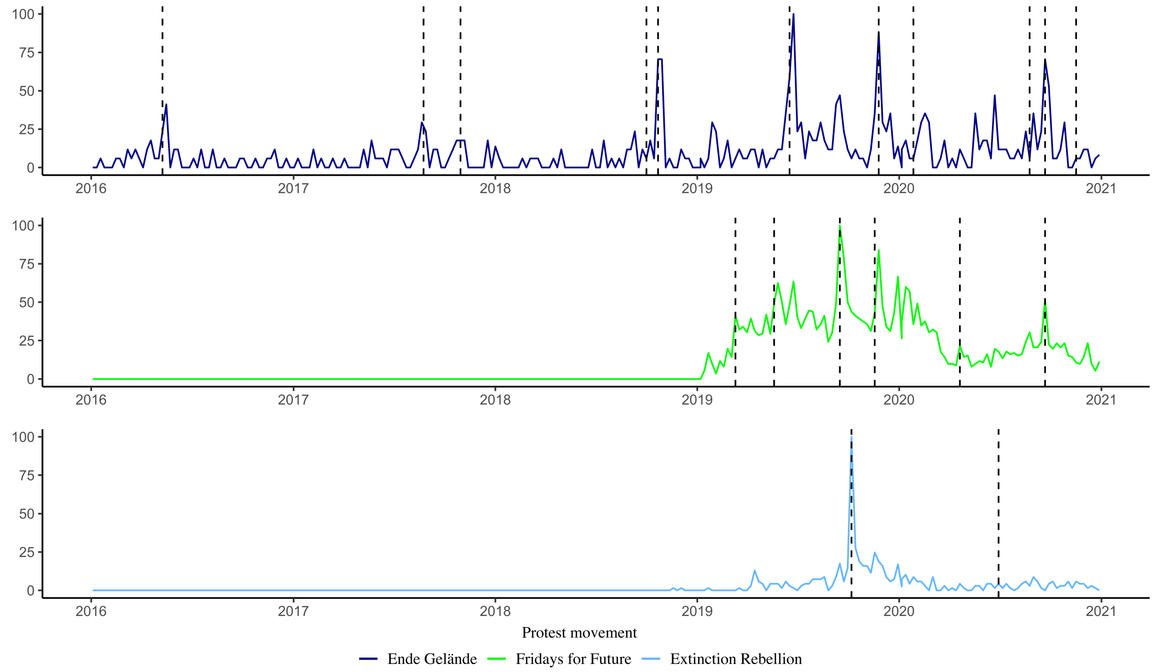

Supplementary Fig. 1 – Number of articles on protest movements in the six highest-circulated German daily newspapers. In the graphs, the weekly data points are relative to the week with the most articles on the respective movement (maximum = 100). This maximum amount within the years 2016 to 2020 was 17 for "Ende Gelände", 112 for "Fridays for Future", and 69 for "Extinction Rebellion". The articles were retrieved from Dow Jones Factiva. Vertical lines represent the identified protests on national news. Source data are provided as a Source Data file.

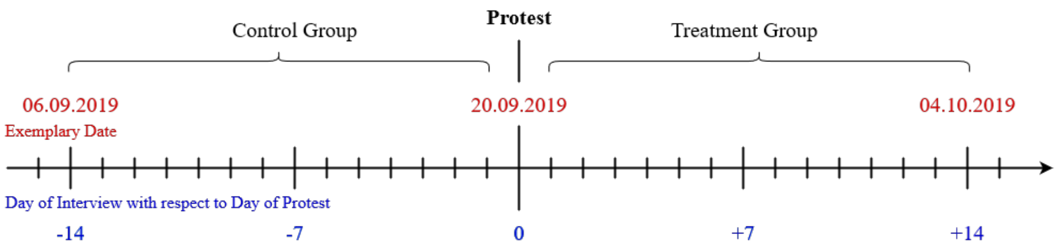

Supplementary Fig. 2 – Exemplary presentation of treatment and control group around a protest. Graphical representation of the main interview time window around an exemplary climate protest.

	Observations	Mean	Estimate	Standard errors	P value
Age of Individual	24,487	47.8106	0.3740	(1.2240)	[0.7639]
Female	24,481	0.5258	0.0100	(0.0063)	[0.1304]
Number of Persons in HH	24,488	2.8324	0.0466	(0.0301)	[0.1404]
Number of Years of Education	23,024	12.4553	0.0118	(0.0873)	[0.8946]
Employment Status of Individual	24,487	0.7049	-0.0083	(0.0238)	[0.7311]
Number of HH-members age 14-18	24,488	0.2578	0.0142	(0.0129)	[0.2848]
HH Labor Income	24,440	55,927.75	1,255.15	(1,480.86)	[0.4092]
Strong interest in politics (2015)	15,626	0.3818	-0.0032	(0.0081)	[0.7025]
Political orientation (2014)	13,663	1.8582	-0.0211	(0.0148)	[0.1721]

Supplementary Tab. 1 – Balance test: treatment and control group. Descriptive statistics of differences of respondents in sociodemographic characteristics in the 14 days before and after the occurrence of a climate protest. Estimates are based on separate regressions of the characteristics on a dummy indicating post- or pre-treatment. The regressions control for a full set of protest fixed effects, ensuring that we compare the average characteristic around each specific protest. We take pre-treatment values of strong interest in politics (2015) and political orientation (2014). Political orientation has the categories left-leaning, center, and right-leaning (categorical variable increasing in this order). Robust standard errors are clustered at the protest level. P values are based on two-tailed t-tests.

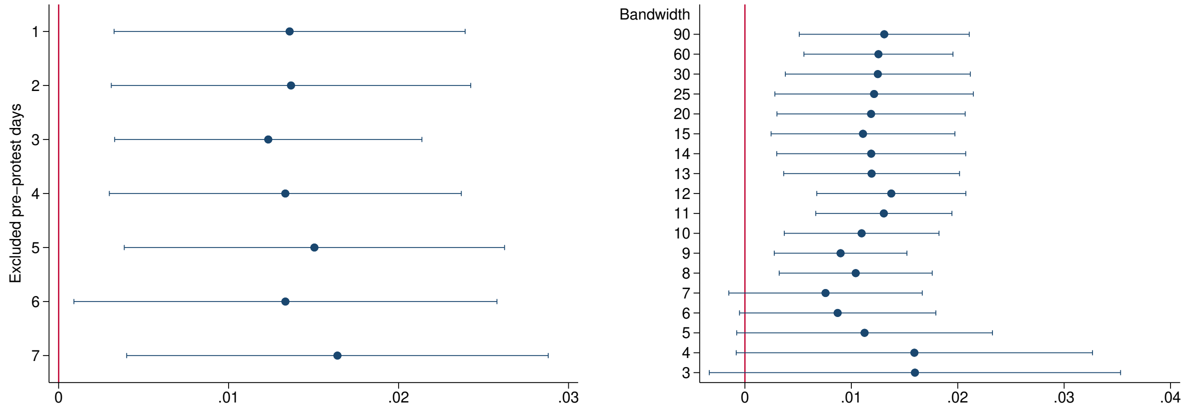

a Excluding Anticipation

b Different Time Windows

Supplementary Fig. 3 – Treatment effects for alternative time windows. **a**, Coefficients and 95 percent confidence intervals excluding up to seven days before a climate protest to account for anticipation. The coefficients are estimated using the most restrictive specification Tab. 2 (Column (6), LPM) and alternating the window around a climate protest. The number of observations ranges from N=23,548 (excluding 1 day) to N=18,109 (excluding 7 days). **b**, Coefficients and 95 percent confidence intervals varying the time window around a climate protest. The number of observations ranges from N=5,306 (3 days) to N=81,202 (90 days). If we decrease the time window around a protest to seven days or less, the estimates become statistically indistinguishable from zero at the five percent significance level due to the loss in the number of observations. Source data are provided as a Source Data file.

**Panel A: Placebo Outcomes**							
	Placebo Concern (0-1 dummy)	(1)	(2)	(3)	(4)	(5)	(6)
Post	General econ. development	0.0097	0.0114	0.0113	0.0121	0.0114	0.0108
		(0.0074)	(0.0093)	(0.0094)	(0.0094)	(0.0091)	(0.0084)
		[0.2077]	[0.2349]	[0.2476]	[0.2169]	[0.2281]	[0.2167]
Number of observations		24,545	24,545	24,520	24,520	24,520	24,514
Post	Own econ. situation	0.0023	0.0131	0.0130	0.0129	0.0127	0.0134
		(0.0097)	(0.0064)	(0.0069)	(0.0068)	(0.0071)	(0.0069)
		[0.8151]	[0.0590]	[0.0773]	[0.0777]	[0.0923]	[0.0714]
Number of observations		31,476	31,476	31,451	31,451	31,451	31,448
Post	Own Pension	0.0066	-0.0007	0.0012	0.0029	0.0027	0.0034
		(0.0059)	(0.0083)	(0.0079)	(0.0076)	(0.0079)	(0.0076)
		[0.2805]	[0.9361]	[0.8801]	[0.7039]	[0.7332]	[0.6592]
Number of observations		24,561	24,561	24,536	24,536	24,536	24,530
Post	Own health	0.0010	-0.0090	-0.0096	-0.0096	-0.0092	-0.0093
		(0.0077)	(0.0042)	(0.0044)	(0.0041)	(0.0045)	(0.0044)
		[0.9010]	[0.0484]	[0.0424]	[0.0328]	[0.0588]	[0.0536]
Number of observations		31,502	31,502	31,478	31,478	31,478	31,475
Post	Peacekeeping	0.0036	0.0009	0.0019	0.0018	0.0017	0.0012
		(0.0067)	(0.0041)	(0.0045)	(0.0046)	(0.0046)	(0.0047)
		[0.5936]	[0.8226]	[0.6804]	[0.6968]	[0.7093]	[0.8084]
Number of observations		24,574	24,574	24,549	24,549	24,549	24,543
Post	Crime	0.0161	0.0124	0.0134	0.0133	0.0123	0.0109
		(0.0103)	(0.0051)	(0.0049)	(0.0048)	(0.0049)	(0.0049)
		[0.1371]	[0.0272]	[0.0143]	[0.0130]	[0.0233]	[0.0423]
Number of observations		24,547	24,547	24,522	24,522	24,522	24,516
Post	Job Security	-0.0018	0.0047	0.0031	0.0050	0.0038	0.0009
		(0.0125)	(0.0101)	(0.0090)	(0.0089)	(0.0081)	(0.0079)
		[0.8899]	[0.6489]	[0.7322]	[0.5863]	[0.6468]	[0.9101]
Number of observations		16,804	16,804	16,786	16,786	16,786	16,779
Post	Immigration	0.0064	-0.0010	-0.0014	-0.0013	-0.0021	-0.0026
		(0.0110)	(0.0059)	(0.0061)	(0.0058)	(0.0060)	(0.0060)
		[0.5676]	[0.8653]	[0.8192]	[0.8198]	[0.7245]	[0.6736]
Number of observations		24,524	24,524	24,499	24499	24,499	24,493
Post	Xenophobia	0.0043	0.0048	0.0056	0.0061	0.0063	0.0037
		(0.0100)	(0.0070)	(0.0070)	(0.0059)	(0.0054)	(0.0062)
		[0.6728]	[0.5008]	[0.4339]	[0.3210]	[0.2585]	[0.5564]
Number of observations		31,406	31,406	31,381	31,381	31,381	31,378
**Panel B: Placebo in time**							
Post		0.0036	0.0031	0.0031	0.0041	0.0031	0.0026
		(0.0038)	(0.0038)	(0.0037)	(0.0038)	(0.0037)	(0.0036)
		[0.3506]	[0.4236]	[0.4016]	[0.2840]	[0.4018]	[0.4838]
Number of observations		80,048	80,048	79,974	79,974	79,974	79,952
Protest FE		✓	✓	✓	✓	✓	✓
Year FE		✓	✓	✓	✓	✓	✓
Individual controls		✓	✓	✓	✓	✓	✓
Month FE			✓	✓	✓	✓	✓
Weekday FE			✓	✓	✓	✓	✓
Weather controls				✓	✓	✓	✓
Elections/COPs controls				✓	✓	✓	✓
Interviewer controls					✓	✓	✓
State FE						✓	✓
Protest×State FE							✓

Supplementary Tab. 2 – Placebo results. a, Effect of climate protests on other concerns that are part of the standard SOEP. **b**, Hypothetical climate protests timed at the 15th of each month, excluding any events with pre- and post-time windows falling into the time windows of actual climate protests. Robust standard errors are clustered at the protest level and in parentheses. P values are based on two-tailed t-tests and in square brackets.

	Dependent variable: Climate concern (0-1 dummy)					
Coefficient	(1)	(2)	(3)	(4)	(5)	(6)
43-56 days before the protest	0.0057	-0.0045	-0.0046	-0.0073	-0.0055	-0.0002
	(0.0053)	(0.0075)	(0.0082)	(0.0078)	(0.0084)	(0.0089)
	[0.2972]	[0.5511]	[0.5850]	[0.3620]	[0.5222]	[0.9803]
29-42 days before the protest	0.0027	-0.0063	-0.0063	-0.0082	-0.0076	-0.0031
	(0.0075)	(0.0101)	(0.0114)	(0.0112)	(0.0105)	(0.0102)
	[0.7249]	[0.5391]	[0.5876]	[0.4754]	[0.4813]	[0.7638]
15-28 days before the protest	0.0051	-0.0018	-0.0014	-0.0016	-0.0019	0.0014
	(0.0049)	(0.0072)	(0.0078)	(0.0078)	(0.0072)	(0.0072)
	[0.3108]	[0.8097]	[0.8581]	[0.8359]	[0.7996]	[0.8516]
1-14 days after the protest	0.0110	0.0150	0.0139	0.0146	0.0147	0.0145
	(0.0058)	(0.0034)	(0.0035)	(0.0036)	(0.0038)	(0.0038)
	[0.0753]	[0.0004]	[0.0011]	[0.0010]	[0.0013]	[0.0017]
15-28 days after the protest	-0.0018	0.0078	0.0032	0.0060	0.0077	0.0089
	(0.0113)	(0.0049)	(0.0051)	(0.0054)	(0.0057)	(0.0056)
	[0.8749]	[0.1332]	[0.5350]	[0.2842]	[0.1998]	[0.1287]
29-42 days after the protest	0.0104	0.0246	0.0213	0.0223	0.0242	0.0286
	(0.0064)	(0.0110)	(0.0099)	(0.0097)	(0.0097)	(0.0094)
	[0.1211]	[0.0400]	[0.0471]	[0.0360]	[0.0235]	[0.0076]
43-56 days after the protest	-0.0003	0.0191	0.0138	0.0168	0.0166	0.0151
	(0.0191)	(0.0234)	(0.0251)	(0.0264)	(0.0281)	(0.0269)
	[0.9895]	[0.4256]	[0.5913]	[0.5329]	[0.5637]	[0.5822]
Protest fixed effects	✓	✓	✓	✓	✓	✓
Year fixed effects	✓	✓	✓	✓	✓	✓
Individual controls	✓	✓	✓	✓	✓	✓
Calendar month fixed effects		✓	✓	✓	✓	✓
Weekday fixed effects		✓	✓	✓	✓	✓
Weather controls			✓	✓	✓	✓
Elections/COPs controls			✓	✓	✓	✓
Interviewer controls				✓	✓	✓
State fixed effects					✓	✓
Protest×State fixed effects						✓
Number of observations	66,990	66,990	66,915	66,915	66,915	66,915

Supplementary Tab. 3 – Treatment effects over time. The presents the results using a linear probability model when extending the time window around a protest to up to 16 weeks and estimating separate treatment effects for each 14-day time interval. The coefficients are relative to the 14 days before a protest. Robust standard errors are clustered at the protest level and in parentheses. P values are based on two-tailed t-tests and in square brackets.

	Dependent variable: Climate concern (0-1 dummy)					
	(1)	(2)	(3)	(4)	(5)	(6)
Post	0.3332	0.1503	0.1622	0.1632	0.1614	0.1370
	(0.1478)	(0.0464)	(0.0521)	(0.0527)	(0.0484)	(0.0461)
	[0.0385]	[0.0051]	[0.0067]	[0.0069]	[0.0042]	[0.0090]
Post × average pre-protest concern	-0.3701	-0.1577	-0.1715	-0.1723	-0.1705	-0.1436
	(0.1666)	(0.0538)	(0.0605)	(0.0611)	(0.0561)	(0.0532)
	[0.0411]	[0.0098]	[0.0119]	[0.0123]	[0.0078]	[0.0158]
Protest FE	✓	✓	✓	✓	✓	✓
Year FE	✓	✓	✓	✓	✓	✓
Individual controls	✓	✓	✓	✓	✓	✓
Month FE		✓	✓	✓	✓	✓
Weekday FE		✓	✓	✓	✓	✓
Weather controls			✓	✓	✓	✓
Elections/COPs controls			✓	✓	✓	✓
Interviewer controls				✓	✓	✓
State FE					✓	✓
Protest×State FE						✓
Number of observations	24,566	24,566	24,541	24,541	24,541	24,535

Supplementary Tab. 4 – Average marginal effects with respect to pre-protest population concern levels. The table presents the results using a linear probability model when interacting the treatment indicator with the average climate change concern in the 14 days before a protest. The estimated interaction effect in Column (6) is the basis for the plotted coefficients in Fig. 3. Robust standard errors are clustered at the protest level and in parentheses. P values are based on two-tailed t-tests and in square brackets.

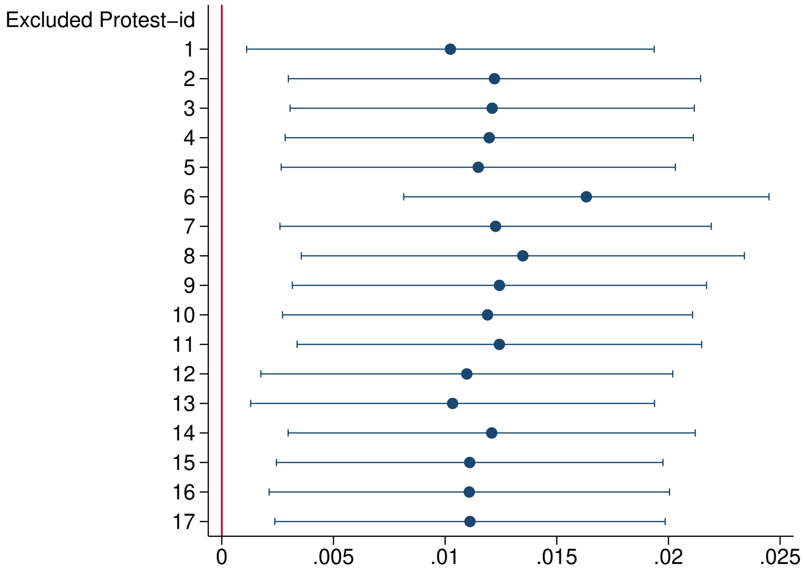

Supplementary Fig. 4 – Iteratively excluding climate protests. The figure plots the coefficients and 95 percent confidence intervals of the treatment effect when iteratively excluding one climate protest at a time. Estimation based on LPM Column (6), Tab. 2. For the number of observations of each iteration subtract the protest-specific N of Supplementary Fig. 6 from N in Tab. 2, Column (6), LPM. Source data are provided as a Source Data file.

	Dependent variable: Climate concern (0-1 dummy)						
	(1)	(2)	(3)	(4)	(5)	(6)	(7)
Post	0.0159	0.0151	0.0082	0.0066	0.0156	0.0302	0.0209
	(0.0046)	(0.0061)	(0.0029)	(0.0071)	(0.0076)	(0.0097)	(0.0054)
	[0.0033]	[0.0259]	[0.0118]	[0.3636]	[0.0563]	[0.0067]	[0.0013]
Post × below median age	-0.0078						
	(0.0063)						
	[0.2309]						
Post × male		-0.0068					
		(0.0083)					
		[0.4252]					
Post × below median income			0.0072				
			(0.0072)				
			[0.3335]				
Post × below median education				0.0132			
				(0.0125)			
				[0.3085]			
Post × pessimistic about future					0.0062		
					(0.0157)		
					[0.6968]		
Post × political orientation: center						-0.0097	
						(0.0168)	
						[0.5722]	
Post × political orientation: left-leaning						-0.0133	
						(0.0133)	
						[0.3317]	
Post × weak or no interest in politics							-0.0161
							(0.0092)
							[0.1014]
Protest FE	✓	✓	✓	✓	✓	✓	✓
Year FE	✓	✓	✓	✓	✓	✓	✓
Individual controls	✓	✓	✓	✓	✓	✓	✓
Month FE	✓	✓	✓	✓	✓	✓	✓
Weekday FE	✓	✓	✓	✓	✓	✓	✓
Weather controls	✓	✓	✓	✓	✓	✓	✓
Elections/COPs controls	✓	✓	✓	✓	✓	✓	✓
Interviewer controls	✓	✓	✓	✓	✓	✓	✓
State FE	✓	✓	✓	✓	✓	✓	✓
Protest×State FE	✓	✓	✓	✓	✓	✓	✓
Number of observations	24,481	24,475	24,434	23,017	14,310	13,642	24,504

Supplementary Tab. 5 – Effects by population subgroup. The table presents the results using a linear probability model when interacting the treatment indicator with respective population indicators. The estimated main and interaction effects are the basis for the plotted coefficients in Fig. 4. Robust standard errors are clustered at the protest level and in parentheses. P values are based on two-tailed t-tests and in square brackets.

Dependent variable: Climate concern (0-1 dummy)

	Model specification	(1)	(2)	(3)	(4)	(5)	(6)
Post	Linear Probability	0.0119	0.0121	0.0124	0.0126	0.0124	0.0117
		(0.0068)	(0.0047)	(0.0048)	(0.0048)	(0.0046)	(0.0043)
		[0.1003]	[0.0217]	[0.0224]	[0.0199]	[0.0171]	[0.0161]
Protest FE		✓	✓	✓	✓	✓	✓
Year FE		✓	✓	✓	✓	✓	✓
Individual controls		✓	✓	✓	✓	✓	✓
Month FE			✓	✓	✓	✓	✓
Weekday FE			✓	✓	✓	✓	✓
Weather controls				✓	✓	✓	✓
Elections/COPs controls				✓	✓	✓	✓
Interviewer controls					✓	✓	✓
State FE						✓	✓
Protest×State FE							✓
Number of observations		23,474	23,474	23,460	23,460	23,460	23,455

Supplementary Tab. 6 – Excluding double protests. The table presents the results using a linear probability model when we exclude the protests at which two separate protests happened simultaneously (within 3 days of each other). Robust standard errors are clustered at the protest level and in parentheses. P values are based on two-tailed t-tests and in square brackets.

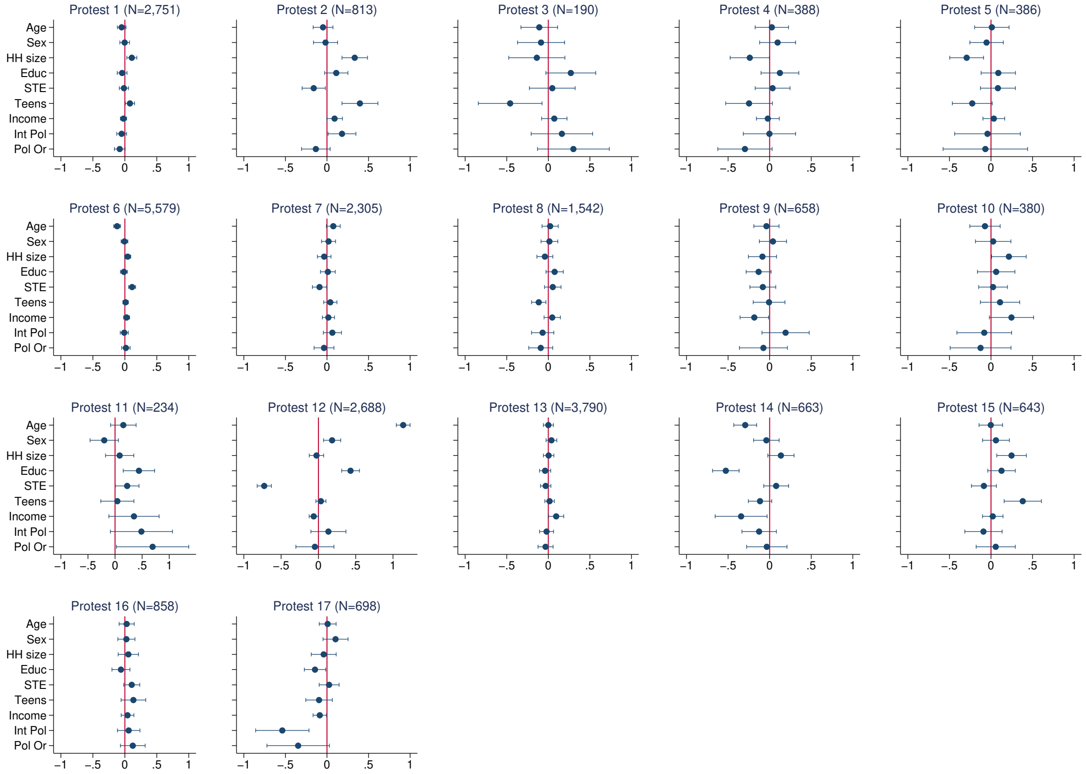

a Balance by protest, no weights

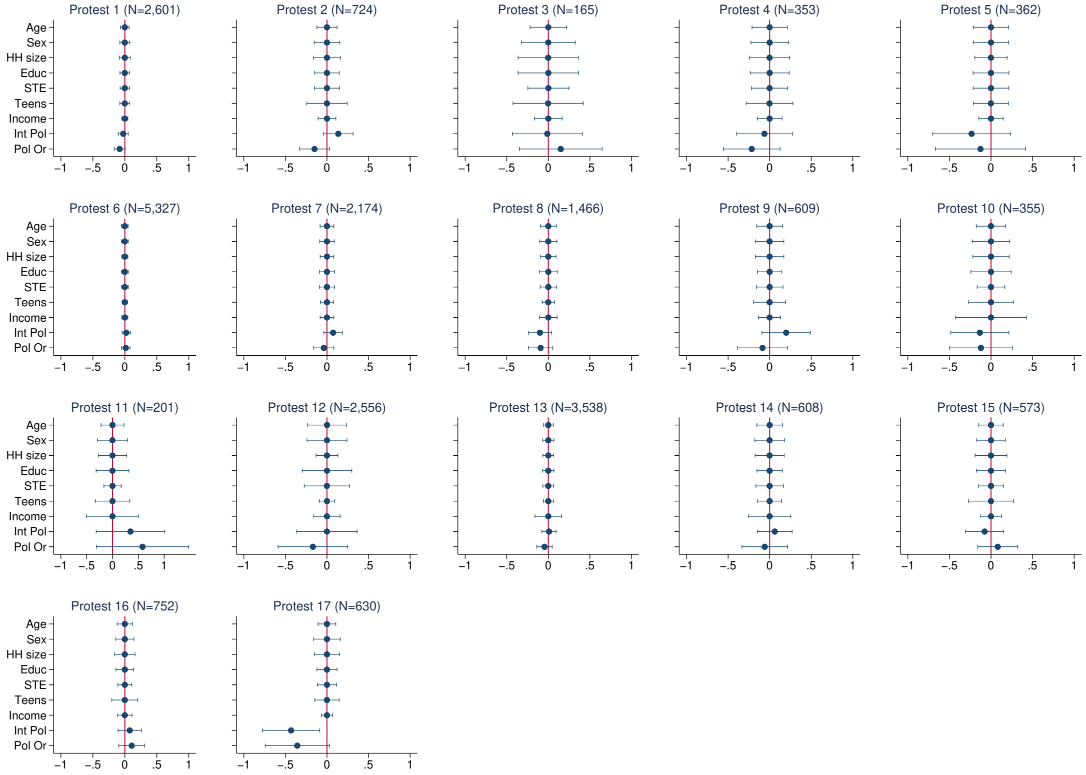

b Balance by protest, entropy balancing weights

Supplementary Fig. 5 – Balance test: treatment and control group by protest. **a** Without weights **b**, With entropy balancing weights. The figure plots the coefficients and 95 percent confidence intervals when regressing the balancing covariates on the treatment indicator. See Supplementary Tab. 1 for details of variables used for balancing. We standardize the covariates to display them in one figure. Source data are provided as a Source Data file.

Protest	General econ. development	Own econ. situation	Own Pension	Own health	Peacekeeping	Crime	Job Security	Immigration	Xenophobia
Protest 1	0.0267	-0.0119	-0.0023	-0.0183	0.0012	0.0141	0.0136	-0.0258	0.0131
	(0.0189)	(0.0192)	(0.0190)	(0.0195)	(0.0115)	(0.0147)	(0.0244)	(0.0166)	(0.0141)
	[0.1568]	[0.5362]	[0.9017]	[0.3481]	[0.9169]	[0.3387]	[0.5782]	[0.1211]	[0.3529]
	2,599	2,628	2,597	2,629	2,602	2,599	1,868	2,597	2,621
Protest 2	-0.2305	-0.0798	-0.1477	-0.1549	-0.0270	-0.1745	-0.0533	-0.1775	0.1726
	(0.0980)	(0.1008)	(0.0899)	(0.1021)	(0.0487)	(0.0582)	(0.1140)	(0.0910)	(0.0681)
	[0.0190]	[0.4288]	[0.1006]	[0.1299]	[0.5790]	[0.0028]	[0.6405]	[0.0516]	[0.0115]
	715	720	721	725	725	725	571	721	718
Protest 3	-0.3451	0.0286	-0.3774	-0.1027	-0.0044	-0.2147	0.2397	-0.2865	-0.0301
	(0.1837)	(0.0942)	(0.1696)	(0.1024)	(0.0789)	(0.1442)	(0.1963)	(0.1982)	(0.0844)
	[0.0631]	[0.7612]	[0.0283]	[0.3164]	[0.9553]	[0.1396]	[0.2259]	[0.1514]	[0.7212]
	163	538	162	539	162	163	135	162	534
Protest 4	0.0590	0.0767	-0.0105	0.0458	0.0314	0.1042	0.1048	0.0407	-0.0779
	(0.0713)	(0.0422)	(0.0621)	(0.0449)	(0.0582)	(0.0682)	(0.0869)	(0.0696)	(0.0413)
	[0.4085]	[0.0695]	[0.8655]	[0.3086]	[0.5904]	[0.1277]	[0.2294]	[0.5591]	[0.0593]
	351	932	351	935	353	352	255	352	929
Protest 5	-0.0297	-0.0157	0.0286	0.0113	-0.0511	-0.0454	0.1111	-0.0818	-0.0252
	(0.0970)	(0.0443)	(0.0939)	(0.0509)	(0.0664)	(0.0722)	(0.1144)	(0.0797)	(0.0452)
	[0.7597]	[0.7240]	[0.7608]	[0.8242]	[0.4424]	[0.5301]	[0.3331]	[0.3057]	[0.5767]
	361	1021	363	1,024	363	363	239	364	1,025
Protest 6	-0.0027	0.0217	0.0065	-0.0046	-0.0084	0.0094	-0.0071	0.0074	0.0161
	(0.0120)	(0.0132)	(0.0126)	(0.0121)	(0.0069)	(0.0099)	(0.0153)	(0.0119)	(0.0083)
	[0.8227]	[0.1006]	[0.6049]	[0.7039]	[0.2228]	[0.3439]	[0.6426]	[0.5358]	[0.0527]
	5,321	5,329	5,329	5,324	5,326	5,327	3,453	5,323	5,322
Protest 7	-0.0176	-0.0095	-0.0332	0.0155	0.0034	-0.0092	-0.0021	0.0047	0.0192
	(0.0239)	(0.0269)	(0.0260)	(0.0254)	(0.0143)	(0.0207)	(0.0307)	(0.0250)	(0.0184)
	[0.4624]	[0.7227]	[0.2022]	[0.5424]	[0.8130]	[0.6565]	[0.9447]	[0.8519]	[0.2965]
	2,172	2,177	2,172	2,175	2,175	2,172	1,530	2,171	2,172
Protest 8	-0.0501	-0.0101	-0.0371	0.0091	0.0234	-0.0006	-0.0069	-0.0093	-0.0169
	(0.0278)	(0.0308)	(0.0295)	(0.0291)	(0.0169)	(0.0240)	(0.0335)	(0.0291)	(0.0221)
	[0.0720]	[0.7427]	[0.2090]	[0.7544]	[0.1646]	[0.9795]	[0.8373]	[0.7498]	[0.4445]
	1,465	1,470	1,470	1,471	1,470	1,466	1,030	1,464	1,468
Protest 9	0.0907	0.0368	0.0176	0.0172	0.0095	-0.0643	0.0244	0.0022	0.0522
	(0.0374)	(0.0307)	(0.0413)	(0.0318)	(0.0265)	(0.0405)	(0.0457)	(0.0429)	(0.0268)
	[0.0156]	[0.2306]	[0.6690]	[0.5886]	[0.7195]	[0.1129]	[0.5935]	[0.9588]	[0.0515]
	606	1,121	606	1,122	607	607	493	607	1,119
Protest 10	-0.0346	-0.0548	-0.1217	-0.0722	0.0189	0.0240	0.0259	-0.1042	-0.0003
	(0.0591)	(0.0427)	(0.0662)	(0.0427)	(0.0455)	(0.0608)	(0.0654)	(0.0700)	(0.0371)
	[0.5583]	[0.2001]	[0.0668]	[0.0912]	[0.6770]	[0.6937]	[0.6926]	[0.1381]	[0.9938]
	355	744	355	743	354	355	298	354	741
Protest 11	-0.0902	0.1206	-0.3582	0.0053	-0.1260	0.5011	-0.5702	0.0283	0.2815
	(0.4737)	(0.1324)	(0.5927)	(0.1809)	(0.1394)	(0.3490)	(0.2660)	(0.4272)	(0.1594)
	[0.8493]	[0.3625]	[0.5466]	[0.9768]	[0.3676]	[0.1534]	[0.0346]	[0.9474]	[0.0779]
	203	654	203	654	201	201	165	201	654
Protest 12	0.0928	0.0291	0.1172	0.0496	-0.0775	0.0558	-0.0025	0.1078	0.0880
	(0.0746)	(0.0713)	(0.0695)	(0.0619)	(0.0291)	(0.0591)	(0.0696)	(0.0715)	(0.0655)
	[0.2139]	[0.6835]	[0.0919]	[0.4228]	[0.0078]	[0.3453]	[0.9710]	[0.1316]	[0.1792]
	2,555	2,835	2,553	2,834	2,554	2,552	1,363	2,549	2,826
Protest 13	0.0284	0.0015	0.0130	-0.0005	0.0049	0.0264	-0.0332	-0.0037	0.0083
	(0.0147)	(0.0212)	(0.0210)	(0.0213)	(0.0151)	(0.0195)	(0.0242)	(0.0210)	(0.0154)
	[0.0533]	[0.9432]	[0.5350]	[0.9812]	[0.7472]	[0.1762]	[0.1711]	[0.8588]	[0.5880]
	3,540	3,542	3,538	3,539	3,541	3,538	2,483	3,531	3,529
Protest 14	-0.0292	0.0636	-0.0383	0.0180	0.0500	0.1011	0.1009	-0.0697	0.0195
	(0.0822)	(0.1039)	(0.0936)	(0.0947)	(0.0842)	(0.0856)	(0.1205)	(0.1048)	(0.0866)
	[0.7225]	[0.5406]	[0.6825]	[0.8491]	[0.5525]	[0.2382]	[0.4034]	[0.5064]	[0.8216]
	605	608	607	609	608	606	412	606	604
Protest 15	0.0775	0.0724	0.0122	-0.0847	0.1213	0.0258	0.0506	0.0183	-0.0260
	(0.1035)	(0.0729)	(0.1297)	(0.0840)	(0.0539)	(0.0914)	(0.1386)	(0.1179)	(0.0713)
	[0.4542]	[0.3207]	[0.9253]	[0.3139]	[0.0248]	[0.7780]	[0.7150]	[0.8766]	[0.7159]
	574	1,012	574	1,015	575	572	464	572	1,012
Protest 16	0.0599	0.0470	0.1117	0.0074	0.0096	0.0395	0.0933	0.0201	0.0502
	(0.0387)	(0.0338)	(0.0446)	(0.0352)	(0.0311)	(0.0419)	(0.0464)	(0.0447)	(0.0291)
	[0.1220]	[0.1639]	[0.0126]	[0.8332]	[0.7587]	[0.3462]	[0.0451]	[0.6536]	[0.0851]
	747	1,208	748	1,216	748	749	612	747	1,206
Protest 17	0.0270	-0.0048	0.0179	0.0077	0.0099	0.0558	0.0200	0.0157	0.0101
	(0.0427)	(0.0362)	(0.0469)	(0.0390)	(0.0403)	(0.0456)	(0.0515)	(0.0471)	(0.0374)
	[0.5269]	[0.8942]	[0.7026]	[0.8425]	[0.8061]	[0.2216]	[0.6978]	[0.7392]	[0.7861]
	629	913	627	915	625	621	529	624	911
Individual controls	✓	✓	✓	✓	✓	✓	✓	✓	✓
Month FE	✓	✓	✓	✓	✓	✓	✓	✓	✓
Weekday FE	✓	✓	✓	✓	✓	✓	✓	✓	✓
Weather controls	✓	✓	✓	✓	✓	✓	✓	✓	✓
Elections/COPs controls	✓	✓	✓	✓	✓	✓	✓	✓	✓
Interviewer controls	✓	✓	✓	✓	✓	✓	✓	✓	✓
State FE	✓	✓	✓	✓	✓	✓	✓	✓	✓

Supplementary Tab. 7 – Placebo results per protest. Effect of each separate climate protest on placebo concerns. The estimation for Protest 14 does not include month fixed effects since they are collinear with the treatment effect. Coefficients are estimated with a LPM with entropy balancing weights applied. For each protest, the first line states the coefficient, the second robust standard errors in parentheses, the third p values based on two-tailed t-tests in square brackets, and the fourth line the number of observations.

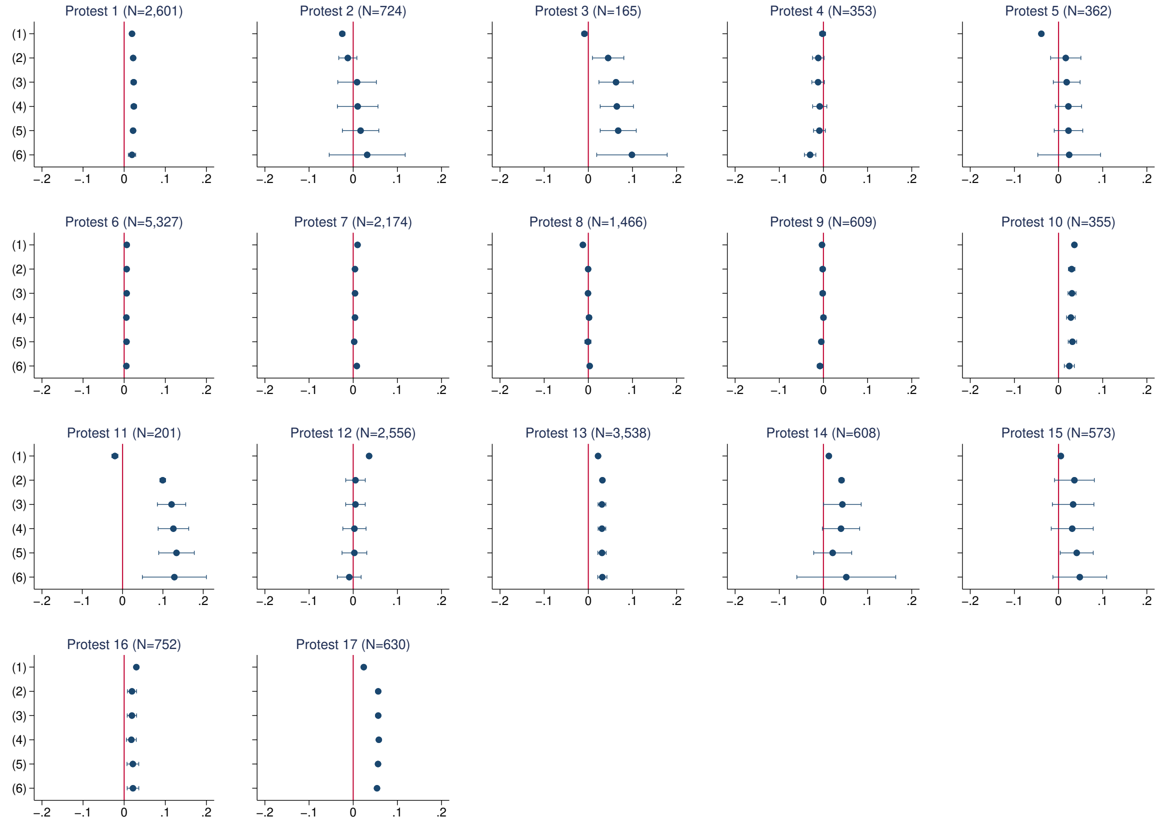

Supplementary Fig. 6 – Main results by individual climate protest. The figure plots the coefficients and 95 percent confidence intervals of individual climate protests on climate change concerns. The numbers in parentheses correspond to the Columns in Tab. 2, LPM model. Entropy balancing weights applied. Source data are provided as a Source Data file.

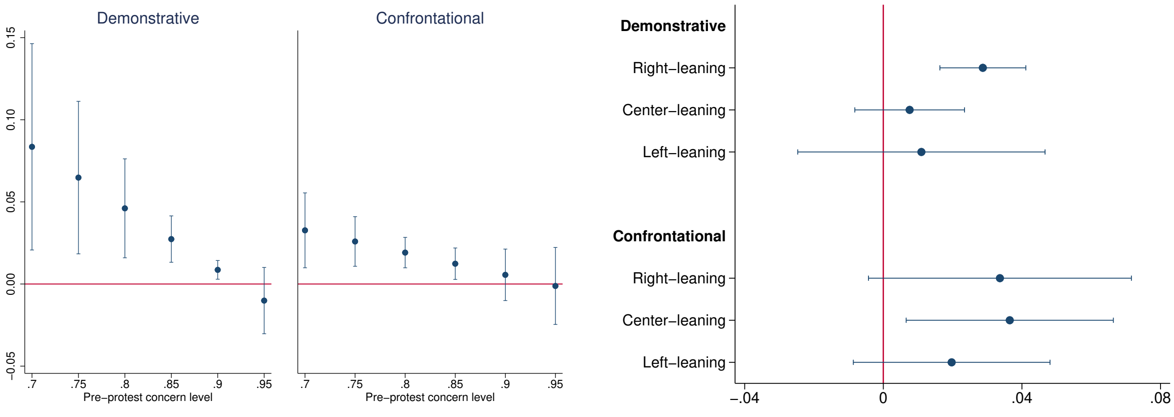

a Effects by pre-protest concern level

b Effects by political orientation

Supplementary Fig. 7 – Effects by dominant protest strategy. **a**, Marginal effects with respect to pre-protest level by dominant protest strategy. The estimation is based on a triple interaction of the treatment effect, the pre-protest concern level in the 14 days before a protest, and the type of protest tactics – either confrontational or demonstrative. **b**, Heterogeneous effects with 95 percent confidence intervals of political orientation by the dominant protest strategies. The estimation is based on a triple interaction of the treatment effect, political orientation, and the type of protest tactics. See Supplementary Tab. 4 and Fig. 4 notes for details on estimation, respectively. Source data are provided as a Source Data file.

	Pr(no concern)	Pr(some concern)	Pr(high concern)	Multinomial logit	Generalized ordered logit
Post	-0.0119	0.0047	0.0072		
	(0.0042)	(0.0079)	(0.0117)		
	[0.0120]	[0.5605]	[0.5460]		
Marginal effect on no concern				-0.0122	-0.0124
				(0.0038)	(0.0041)
				[0.0013]	[0.0027]
Marginal effect on some concern				0.0053	0.0058
				(0.0084)	(0.0083)
				[0.5245]	[0.4887]
Marginal effect on high concern				0.0069	0.0066
				(0.0116)	(0.0116)
				[0.5530]	[0.5700]
Protest FE	✓	✓	✓	✓	✓
Year FE	✓	✓	✓	✓	✓
Individual controls	✓	✓	✓	✓	✓
Month FE	✓	✓	✓	✓	✓
Weekday FE	✓	✓	✓	✓	✓
Weather controls	✓	✓	✓	✓	✓
Elections/COPs controls	✓	✓	✓	✓	✓
Interviewer controls	✓	✓	✓	✓	✓
State FE	✓	✓	✓	✓	✓
Protest×State FE	✓	✓	✓	✓	✓
Number of observations	24,535	24,535	24,535	24,541	24,541

Supplementary Tab. 8 – Intensive margin. Columns (1) to (3) are separate regressions where the outcome is coded as a dummy variable indicating 1 for the respective concern level and 0 otherwise. Column (4) represents the marginal effects from a joint estimation using a multinomial logit model. Column (5) represents the marginal effects from a generalized ordered logit model. We choose the generalized ordered logit over the ordered logit model since the proportional odds assumption is likely violated. Columns (4) and (5) do not control for household size, the number of children aged 14 to 18 in the household, and employment status for computational reasons. Robust standard errors are clustered at the protest level and in parentheses. P values are based on two-tailed t-tests and in square brackets.

	Related concern (0-1 dummy)	(1)	(2)	(3)	(4)	(5)	(6)
Post	Environmental protection	0.0108	0.0097	0.0099	0.0102	0.0100	0.0090
		(0.0054)	(0.0042)	(0.0043)	(0.0043)	(0.0043)	(0.0041)
		[0.0612]	[0.0354]	[0.0361]	[0.0325]	[0.0316]	[0.0441]
Protest FE		✓	✓	✓	✓	✓	✓
Year FE		✓	✓	✓	✓	✓	✓
Individual controls		✓	✓	✓	✓	✓	✓
Month FE			✓	✓	✓	✓	✓
Weekday FE			✓	✓	✓	✓	✓
Weather controls				✓	✓	✓	✓
Elections/COPs controls				✓	✓	✓	✓
Interviewer controls					✓	✓	✓
State FE						✓	✓
Protest×State FE							✓
Number of observations		24,583	24,583	24,558	24,558	24,558	24,552

Supplementary Tab. 9 – Effect of climate protests on concerns about the environment. Coefficients are estimated using a LPM. Robust standard errors are clustered at the protest level and in parentheses. P values are based on two-tailed t-tests and in square brackets.
