# Supplementary material for: Increase in concerns about climate change following climate strikes and civil disobedience in Germany
Source: Nat Commun. 2024 Apr 4;15:2916. doi: 10.1038/s41467-024-46477-4 (PMC10995135; doi:10.1038/s41467-024-46477-4)
Supplement: Supplementary file 3 — Reporting Summary [file 41467_2024_46477_MOESM3_ESM.pdf]

Corresponding author(s): Johannes Brehm, Henri Gruhl

Last updated by author(s): 2024/01/03

Reporting Summary

Nature Portfolio wishes to improve the reproducibility of the work that we publish. This form provides structure for consistency and transparency in reporting. For further information on Nature Portfolio policies, see our Editorial Policies and the Editorial Policy Checklist.

Statistics

For all statistical analyses, confirm that the following items are present in the figure legend, table legend, main text, or Methods section.

n/a	Confirmed	
☐	☒	The exact sample size (*n*) for each experimental group/condition, given as a discrete number and unit of measurement
☐	☒	A statement on whether measurements were taken from distinct samples or whether the same sample was measured repeatedly
☐	☒	The statistical test(s) used AND whether they are one- or two-sided<br>*Only common tests should be described solely by name; describe more complex techniques in the Methods section.*
☐	☒	A description of all covariates tested
☐	☒	A description of any assumptions or corrections, such as tests of normality and adjustment for multiple comparisons
☐	☒	A full description of the statistical parameters including central tendency (e.g. means) or other basic estimates (e.g. regression coefficient) AND variation (e.g. standard deviation) or associated estimates of uncertainty (e.g. confidence intervals)
☐	☒	For null hypothesis testing, the test statistic (e.g. *F*, *t*, *r*) with confidence intervals, effect sizes, degrees of freedom and *P* value noted<br>*Give P values as exact values whenever suitable.*
☒	☐	For Bayesian analysis, information on the choice of priors and Markov chain Monte Carlo settings
☒	☐	For hierarchical and complex designs, identification of the appropriate level for tests and full reporting of outcomes
☒	☐	Estimates of effect sizes (e.g. Cohen's *d*, Pearson's *r*), indicating how they were calculated

Our web collection on statistics for biologists contains articles on many of the points above.

Software and code

Policy information about availability of computer code

Data collection	Software: Stata MP 16 64-bit, R version 4.3.1;<br>Programms: Stata: reghdfe (version 5.7.3), ebalance (version 1.5.4), estout (version 3.17), coefplot (version 1.8.5), mlogit (version 11.4.2), gologit2 (version 3.2.5); R: ggplot2 (version 3.4.3), lubridate (version 1.9.2), readtext (version 0.90), dplyr (version 1.1.2), gridExtra (version 2.3), haven (version 2.5.3), patchwork (version 1.1.3);<br>Custom code: https://github.com/JohannesBrehm/Climate_protests
Data analysis	Software: Stata MP 16 64-bit, R version 4.3.1;<br>Programms: Stata: reghdfe (version 5.7.3), ebalance (version 1.5.4), estout (version 3.17), coefplot (version 1.8.5), mlogit (version 11.4.2), gologit2 (version 3.2.5); R: ggplot2 (version 3.4.3), lubridate (version 1.9.2), readtext (version 0.90), dplyr (version 1.1.2), gridExtra (version 2.3), haven (version 2.5.3), patchwork (version 1.1.3);<br>Custom code: https://github.com/JohannesBrehm/Climate_protests

For manuscripts utilizing custom algorithms or software that are central to the research but not yet described in published literature, software must be made available to editors and reviewers. We strongly encourage code deposition in a community repository (e.g. GitHub). See the Nature Portfolio guidelines for submitting code & software for further information.

Data

Policy information about availability of data

All manuscripts must include a data availability statement. This statement should provide the following information, where applicable:

- Accession codes, unique identifiers, or web links for publicly available datasets
- A description of any restrictions on data availability
- For clinical datasets or third party data, please ensure that the statement adheres to our policy

The following data availability statement is included in the manuscript: "Access to the individual-level data (SOEP-Core v37eu, doi: 10.5684/soep.core.v37eu) can be requested by signing an agreement with DIW Berlin. Source data are provided with this paper. All remaining data to replicate the analysis, tables, and figures can be retrieved from https://github.com/JohannesBrehm/Climate_protests."

Human research participants

Policy information about studies involving human research participants and Sex and Gender in Research.

Reporting on sex and gender	We control for sex (biological attribute) and report heterogeneities by sex. The sex is determined by the SOEP and based on self-reporting.
Population characteristics	See below.
Recruitment	See Goebel, J., Grabka, M. M., Liebig, S., Kroh, M., Richter, D., Schröder, C., & Schupp, J. The German socio-economic panel (SOEP). Jahrbücher für Nationalökonomie und Statistik, 239(2), 345-360 (2019) for a detailed explanation how households were recruited to participate in the SOEP.
Ethics oversight	Ethics approval by Hertie School's Research Ethics Officer under application ID 20230220-27.

Note that full information on the approval of the study protocol must also be provided in the manuscript.

Field-specific reporting

Please select the one below that is the best fit for your research. If you are not sure, read the appropriate sections before making your selection.

☐ Life sciences ☒ Behavioural & social sciences ☐ Ecological, evolutionary & environmental sciences

For a reference copy of the document with all sections, see nature.com/documents/nr-reporting-summary-flat.pdf

Behavioural & social sciences study design

All studies must disclose on these points even when the disclosure is negative.

Study description	The study exploits differences in the timing of survey interviews of the SOEP relative to the occurrence of climate protests to quantitatively measure the causal impact of climate protests on climate change concerns by comparing climate change concerns before and after climate protests controlling for counfounding factors.
Research sample	The main empirical analysis relies on the SOEP (SOEP-Core v37eu), a representative longitudinal household survey of about 15,000 households conducted yearly since 1984 in Germany. The data contain information from all household members aged 12 years and over, which includes information on approximately 30,000 individuals. The survey covers a wide range of topics and socioeconomic characteristics including indicators of attitudes and concerns.The main sample consists of those surveyed in the 14 days before (N=12,633) and after (N=11,933) an identified climate protest. The SOEP is suitable for the chosen method since it surveys individuals across the entire year.
Sampling strategy	"SOEP only uses random probability samples. General population samples typically draw on a nation-wide two-stage stratified sampling procedure (Sample A, C, E, F, H, J, K). First, nation-wide sample points are sampled by federal state and municipality size. To secure efficient face-to-face interviewing, the number of regional sample points ranges between 125 and 985 per sample. Second, within each sample point, households are sampled in a random walk procedure. In refreshment samples F, J, and K, a second stratification stage distinguishes natives and migrants." Goebel, J., Grabka, M. M., Liebig, S., Kroh, M., Richter, D., Schröder, C., & Schupp, J. The German socio-economic panel (SOEP). Jahrbücher für Nationalökonomie und Statistik, 239(2), 345-360 (2019).
Data collection	The individual-level data (SOEP-Core v37eu) was provided by DIW Berlin. Climate protests salient to the general public were included in the protests database when mentioned in the evening news formats of the ARD or ZDF, the two main public broadcasters in Germany. To support the salience of the movements organizing the climate protests in the public, we used Google Trends and the number of newspaper articles in the six highest-circulation newspapers through the Factiva database by Dow Jones & Company. The researchers were not blinded to the study hypothesis.

Timing	We collect climate protests and use SOEP data from 2016 to 2020.
Data exclusions	The effective sample size of all individuals surveys between 2016 and 2020 with a non-missing outcome variable is N=128,851. The sample size in our standard time window of 14 days before and after a protest (see Research sample) is N=24,566. Consequently, we exclude N=104,285 individuals in this time window specification. Our largest time window comprises those individuals surveyed up to 90 days before and after the climate protests (N=81,293). Here, we exclude N=47,558 individuals from the data.
Non-participation	N=714 individuals do not respond to the outcome variable from 2016 to 2020. This corresponds to a non-response rate of 0.6 percent. General population refreshments are integrated regularly to the SOEP to combat attrition and maintain representativeness. During our study period a refreshment sample was added in 2017 (N=2,314).
Randomization	Participants were allocated to the control group, if they were interviewed in the specified time window before a climate protests and allocated the to the treatment group, if they were interviewed in the specified time window after a climate protest.

Reporting for specific materials, systems and methods

We require information from authors about some types of materials, experimental systems and methods used in many studies. Here, indicate whether each material, system or method listed is relevant to your study. If you are not sure if a list item applies to your research, read the appropriate section before selecting a response.

Materials & experimental systems

n/a	Involved in the study
☒	☐ Antibodies
☒	☐ Eukaryotic cell lines
☒	☐ Palaeontology and archaeology
☒	☐ Animals and other organisms
☒	☐ Clinical data
☒	☐ Dual use research of concern

Methods

n/a	Involved in the study
☒	☐ ChIP-seq
☒	☐ Flow cytometry
☒	☐ MRI-based neuroimaging
